# The influence of fetal sex on antenatal maternal glucose and insulin dynamics

**DOI:** 10.3389/fcdhc.2024.1351317

**Published:** 2024-12-17

**Authors:** Thomas P. Mullins, Linda A. Gallo, H. David McIntyre, Helen L. Barrett

**Affiliations:** ^1^ Mater Research Institute, The University of Queensland, Brisbane, QLD, Australia; ^2^ School of Health, University of the Sunshine Coast, Petrie, QLD, Australia; ^3^ Mater Research Institute, The University of Queensland, and Mater Hospital Brisbane, Brisbane, QLD, Australia; ^4^ Obstetric Medicine, Royal Hospital for Women, Randwick and Medicine at The University of New South Wales, Sydney, NSW, Australia

**Keywords:** gestational diabetes mellitus, fetal sex, maternal metabolism, mechanism, glucose, insulin

## Abstract

The ‘Developmental Origins of Health and Disease’ (DOHaD) hypothesis postulates that exposures during critical periods of development and growth, including maternal hyperglycemia, can have significant consequences for short- and long-term health in offspring. The influence of fetal status on maternal (patho)physiology is less well understood but gaining attention. Fetal sex specifically may be an independent risk factor for a range of adverse pregnancy outcomes, including increased gestational diabetes mellitus (GDM) frequency with male fetuses in multi-ethnic populations. Fetal sex has been thought to modulate maternal glucose metabolism, including insulin dynamics, through complex genetic and hormonal interactions. Mechanisms have not been fully elucidated, however, but may relate to sexual dimorphism in maternal-fetal-placental interactions. We review current evidence on the potential influence of fetal sex on maternal glucose and insulin dynamics, and fetal outcomes.

## Heterogeneity (spectrum) of gestational diabetes mellitus pathophysiology

The predominant drivers of hyperglycemia in nonpregnant individuals have been identified as either insulin secretion and/or insulin sensitivity defects. There is evidence of a heterogeneity of subtypes driving the GDM pathophysiology. In pregnant women with GDM, Powe et al. (2016) identified that ~51% had a predominant insulin sensitivity defect, ~30% had a predominant insulin secretion defect and remaining ~19% had a combination of both (1). These GDM subtypes appear to have distinct physiology as evident by differences in adipokines, BMI and risk of adverse pregnancy outcomes such as macrosomia, where women with a predominant secretion defect have a greater risk of adverse outcomes. A second study by Layton et al. (2018) further demonstrated that women with GDM-sensitivity defects had increased triglycerides and decreased high density lipoprotein (HDL) compared to women with GDM-secretion defects (2). However, these studies are not without limitations, including a predominantly Caucasian cohort and women with GDM receiving treatment which potentially confounds interpretation of pregnancy outcomes.

An alternative approach taken by Madsen et al. (2021) was the assessment of insulin secretion and sensitivity as continuous variables in relation to pregnancy outcomes (3). The reasoning behind this type of analysis relates to the hyperbolic relationship between insulin sensitivity and secretion in women with and without GDM. Madsen et al. (2021) suggested women with insulin sensitivity defects who have corresponding augmented insulin secretion may remain normoglycemic (3). The study demonstrated that the measurement of insulin secretion and sensitivity as continuous variables significantly improved prediction of adverse pregnancy outcomes compared with dichotomous GDM classifications. Another strength of this study was the removal of the confounding effect of GDM treatment as the cohort were part of the blinded HAPO (Hyperglycemia and Adverse Pregnancy Outcome) study. The hallmark HAPO study came to define new GDM diagnosis criteria and provides valuable insights as participants themselves were not diagnosed as or treated for having GDM.

Taken together, these studies impress the significance of understanding the underlying pathophysiology related to the clinical hyperglycemia. Therefore, understanding the risk factors which contribute, such as fetal sex, is important for assisting in pregnancy-related treatment decisions.

## Fetal and placental influence on maternal physiology

The placenta acts as the critical messenger and conduit between the maternal and fetal environments during gestation. Maternal complications arising in the latter half of gestation have been related to exposures beginning as early as placental implantation. This period is characterized by fetal trophoblast cell invasion into the maternal decasualized endometrium and myometrium as well as the maternal uterine vasculature. This process establishes the definitive uteroplacental circulation initiating the maternal-fetal-placental interplay (4). The mature chorioallantoic placenta provides a vascular connection for acquiring nutrients and oxygen from the maternal circulation and, in return, the placenta synthesizes hormones and cytokines which are imperative for a healthy pregnancy. The secretion of these molecules into the maternal circulation is one mechanism by which the fetal unit influences maternal physiology (5). Previous literature has found high maternal serum human chorionic gonadotrophin levels are associated with both pregnancy-induced hypertension and GDM (6). Additionally, decreases in human placental lactogen and prolactin concentrations have been associated with increased maternal glucose metabolism and fetal weight; the latter likely influenced by the consequent changes to maternal glucose metabolism (5, 7). It’s proposed that imprinted genes, which are expressed from one parental allele due to germline epigenetic changes, may impact placental hormone production by regulating placental lineages responsible for these hormones (8). One such example is the delta-like homolog 1 (DLK1) gene, a product of an imprinted gene mainly expressed from the paternally inherited chromosome during fetal development (9). This gene encodes a single-pass transmembrane protein that, when cleaved, results in a soluble form circulating at high concentrations in maternal blood during late pregnancy, affecting maternal fasting and intrauterine growth (10, 11). In mice lacking fetal-derived DLK1, maternal ketone levels are diminished, and glucose levels are elevated despite normal insulin levels during periods of fasting, which suggests that the switch from glucose to fatty acid oxidation has not occurred (9). In the fed state, pregnant mice without DLK1 do not experience an equivalent magnitude decrease in HDL levels compared to mice with circulating DLK1 (9). Together these data propose that failure to increase DLK1 levels during pregnancy prevent normal maternal metabolic adaptations, specifically reduced HDL levels, and accelerated response to starvation. It is plausible that atypical imprinting in the placenta is one mechanism of how the fetal-placental unit influences maternal physiology.

The complications which arise throughout gestation are a complex interaction between the fetal and maternal physiology which demonstrate sex-specific characteristics. In an elegant study, Takimoto and colleagues established a model of gestational hypertension by mating transgenic females expressing human angiotensinogen with transgenic males expressing human renin (12). The transient rise in maternal blood pressure returned to normal after birth, suggesting the hypertension was driven by the secretion of placental renin into the maternal circulation. In comparison, pregnant female mice derived from other mating combinations did not display signs of pregnancy-induced hypertension. It has been recognized that women carrying a fetus with Beckwith-Wiedemann syndrome have a more than double risk of developing gestational hypertension and impaired glucose tolerance versus carrying a non-affected sibling (13). This effect is not only seen in rare syndromes, but also in more common gene polymorphisms. An example of which is the maternal PROGINS progesterone receptor gene polymorphism which has a frequency range from 0.82 to 0.93 among healthy women. Hocher and colleagues demonstrated the polymorphism alone within the population is not associated with adverse cardiometabolic insults at delivery (14). However, genetically susceptible pregnant women who carry a male fetus report significantly elevated total glycated hemoglobin compared to matched genotype women carrying a female fetus (14).

## Maternal-fetal-placental interplay

Earlier research examining the placenta considered it as an asexual organ, and consequently initial placental studies did not consider the sex. Although its extraembryonic origin is acknowledged, the placenta is now given the sex of the embryo to which it belongs, and sexual dimorphic features are evident throughout placental development (15). There is increasing evidence to suggest sex-specific gene expression may modulate the placenta response to environmental stimuli and influence fetal developmental programming and maternal adaptations during pregnancy (15).

Sex chromosomes are the foundation of determining sex characteristics. In females, one X chromosome is randomly inactivated during embryogenesis and will remain inactivated from parent cell to daughter cells during division. This process is to compensate for receiving both maternal and paternal X chromosomes and thus only allowing one copy to be transcribed. It is suggested that non-random X inactivation can occur in the presence of genetic mutations in one X chromosome or for a selective advantage (16). This means that the female fetus may provide a survival advantage due to the capability of inducing preferential silencing, leading to a biased expression of X-linked genes. The differentiated expression can result in a more favorable adaption to the adverse environment (16). Furthermore, the placenta is the only known tissue to globally reactivate the inactive X chromosome at specific gestational timepoints in females (16). This underscores the potential for the human placenta in females to trigger non-random X inactivation in an adverse intrauterine environment, particularly in the early stages of implantation.

Immunological programming at *the* level of the fetal-placental interface has been found to present with sex specific differences. The Alabama Preterm Birth Study reported placentae of live males delivered less than 32 weeks have significantly higher rates of chorionic inflammatory lesions with greater lymphoplasmacytic cell infiltration compared to preterm female fetuses (17). Additionally, Yeganegi et al. (2009) demonstrated a greater synthesis of active prostaglandins and cytokine expression in male placentae in response to lipopolysaccharides which may provide insight into the higher incidence of male preterm birth (18). Importantly, the regulation of apoptosis, prostaglandin synthesis, vascular permeability and programming of the fetal immune system is driven by the placental immune system and therefore all these pathways may be sexually dimorphic.

Characterization of placental gene expression in non-pathological term pregnancies have found sex-biased patterns which are not observed in other tissues. Buckberry et al. (2014) detected a higher female expression of genes involved in placental development, pregnancy maintenance and maternal immune tolerance of the conceptus (19). On the other hand, the male fetus placenta was found to augment signaling pathways towards an inflammatory state, supporting evidence of a distinct male bias towards placental dysfunction. Currently, however, there is a lack of literature on whether fetal sex impacts on maternal insulin and glucose physiology.

## Fetal sex and pre-existing diabetes mellitus

An initial investigation into sex differences in infant morbidity and mortality associated with maternal pre-existing diabetes found a male disadvantage (20). Specifically, male infants had higher rates of neonatal hypoglycemia and spent more time in neonatal intensive care. Later studies by Evers et al. (2009) and Gracia-Patterson et al. (2011) confirmed a higher incidence of adverse outcomes in male infants of women with pre-existing diabetes (21, 22). In the latter study, there was a significantly higher occurrence of congenital malformations in male newborns (21). The authors suggested this might be linked with the male embryos increased vulnerability to oxidative stress. Persson & Fadl (2014) conducted a large population-based study (n= 13,106 pregnancies) and found that, women with type 1 diabetes, male infants had higher rates of acute respiratory disorders, which was accredited to slower lung maturation, and a tendency to have higher rates of almost all adverse outcomes (23). Whether this increased risk in male fetuses is associated with sex-specific alterations in maternal hyperglycemia and/or other metabolic factors remains unclear from these studies. There was, however, no sex differences in relation to perinatal death, major malformations, or preterm delivery in pregnancies complicated by type 1 or type 2 diabetes. The lack of findings in the type 2 diabetes cohort was suggested to be related to low sample size (n = 412).

## Fetal sex and GDM

A systematic review published by Broere-Brown et al. (2020) included 28 studies comprising more than two million participants and examined the prevalence of GDM related to fetal sex (24). The pooled meta-analysis found women carrying a male fetus compared to women carrying a female fetus had a higher risk of developing GDM (RR: 1.04; 95% CI 1.02-1.07) (24). These findings agree with an earlier systematic review and meta-analysis published by Jaskolka et al. (2015) which confirmed, in a sensitivity analysis of high-quality studies, a male fetus predominance in GDM pregnancies (RR: 1.03; 95% CI 1.01-1.06) (25). Many of the individual studies in these meta-analyses found no association between sex and GDM (**Table 1**). However, this might be due to the lack of granular patient level data provided by large retrospective hospital audits. Broere-Brown and colleagues estimated that 225,000 cases of GDM per year are associated, to some degree, with fetal sex and therefore the overall observed effect could be considered relatively mild (24). Additionally, GDM complicated pregnancies appear to have fetal sex dependent effects on perinatal outcomes. Findings indicate male newborns of GDM pregnancies have increased risk of preterm premature rupture of membranes, neonatal infection, acute respiratory disorders and major malformations (23, 26).

**Table 1:** Studies examining fetal influence on maternal glucose and insulin metabolism and associated risk of GDM.

Study	Sample size	Maternal insulin resistance (male vs female)	Maternal insulin secretory dysfunction (male vs female)	Maternal glucose (male vs female)	Fetal sex association with GDM
Engel et al. (2008) (27)	16,445	n.d	n.d	n.d	ns
Hou et al. (2014) (28)	109,722	n.d	n.d	n.d	ns
Favilli et al. (2013) (29)	623	n.d	n.d	n.d	ns
Liu et al. (2017) (30)	65,173	n.d	n.d	n.d	Male
Sheiner et al. (2004) (31)	108,995	n.d	n.d	n.d	Male
Verburg et al. (2015) (32)	574,358	n.d	n.d	n.d	Male
Khalil & Alzahra (2013) (33)	29,140	n.d	n.d	n.d	Male
Walsh et al. (2015) (34)	582	↔ HOMA-IR	↓ C-peptide	n.d	n.d
Retnakaran & Shah (2015) (35)	642,987	n.d	n.d	n.d	Male
Giannubilo et al. (2018) (36)	327	n.d	n.d	↑ AUC-OGTT	n.d
Yamashita et al. (2020) (37)	617	↓ HOMA-IR	↑ Insulin sensitivity index ↓ Fasting immunoreactive insulin ↔ HOMA-β	↔ Fasting plasma glucose ↔ HbA1c (%)	ns
Aibar et al. (2012) (38)	29,530	n.d	n.d	n.d	Male
Peled et al. (2013) (39)	545	n.d	n.d	n.d	ns
Cosson et al. (2016) (40)	20,149	n.d	n.d	n.d	ns
Ehrlich et al. (2012) (41)	285,529	n.d	n.d	n.d	ns
Retnakaran et al. (2015) (42)	1,074	↔ HOMA-IR	↓ Insulinogenic index ↔ Matsuda index	↑ AUC-OGTT	Male
Xiao et al. (2014) (43)	299	n.d	↓ Insulin ↓ Proinsulin ↑ Insulin sensitivity (glucose-to-insulin ratio)	↔ AUC-OGTT	ns
Ricart et al. (2009) (44)	9,270	n.d	n.d	n.d	ns
Geng et al. (2017) (45)	877	↔ HOMA-IR ↔ Disposition index	↓ HOMA-β ↓ Stumvoll first-phase index ↔ Insulinogenic index ↔ Matsuda index	↑ Fasting plasma glucose ↔ HbA1c (%)	n.d
Petry et al. (2022) (46)	813	↓ HOMA-IR	↑ QUICKI ↔ HOMA-β ↔ Insulinogenic index ↔ Insulin disposition index ↓ fasting insulin	↓ Fasting plasma glucose ↔ AUC-OGTT	ns
Hooks et al. (2023) (47)	43,727	n.d	n.d	n.d	ns
Seghieri et al. (2022) (48)	170,126	n.d	n.d	n.d	Male

Direction of change is in reference to women carrying a male fetus compared to women carrying a female fetus.

ns, non-significant; n.d, no data; HOMA-IR, homeostatic model assessment of insulin resistance; HOMA-β, beta-cell function; HbA1c, glycosylated hemoglobin; QUICKI, quantitative insulin sensitivity check index.

↑, Increase; ↓, Decrease; ↔, No change.

## Fetal sex and maternal glucose and insulin dynamics

The underlying mechanism(s) linking fetal sex with maternal glucose metabolism have not been fully elucidated, however interactions between placental hormones and maternal β-cell function have been postulated. The lack of granular patient level data within this field creates problems when suggesting mechanisms of action as many studies in the field are retrospective hospital audits and consequently have not included information regarding maternal insulin dynamics. The few studies which have data on maternal metabolism suggest there is an association with fetal sex and maternal β-cell function. In a study of women with normoglycemia it was shown that carrying a male fetus was independently associated with increased maternal fasted plasma glucose and decreased β-cell function as measured by HOMA-β (45). To expand on this population, Retnakaran et al. (2015) observed an independent effect of male sex on reduced HOMA-β and increased plasma glucose at 29 weeks’ gestation in both women with normoglycemia or GDM (42). A following study found women with GDM carrying a male fetus required more frequent insulin therapy which stands to reason when interpreted with the decrease in β-cell function (36).

There are no studies at the time of this review which have found that carrying a female fetus increases the maternal risk of GDM. However, there is evidence to suggest that carrying a female fetus independently increases maternal insulin resistance (37, 43). Xiao et al. (2014) and Yamashita et al. (2020) reported that carrying a female fetus was associated with augmented maternal insulin levels and corresponding insulin resistance during gestation (37, 43). Neither study observed changes in HOMA-β and frequency of GDM. However, any sub-clinical effects of sex-specific insulin dysregulation could have short- and long-term implications for both mother and baby. For example, Retnakaran & Shah (2015) suggest first-time pregnant women with GDM have an increased likelihood of developing type 2 diabetes mellitus postpartum if they gave birth to a girl (35). The authors of the paper postulate women with GDM carrying a female reflects poorer β-cell function prior to pregnancy rather than the effect of placental hormones contributing to maternal metabolic health observed in women with GDM carrying a male. However, neither β-cell function nor insulin resistance were measured in Retnakaran & Shah (2015), thus the underlying basis for increased type 2 diabetes risk following GDM pregnancies carrying a female warrants further research (35).

Comparing or extrapolating these data to the general population becomes challenging due to variations in average body mass index, prevalence of glucose metabolism disorders among women, and variable use of insulin resistance and secretion metrics. Therefore, a clinical dataset incorporating large and ethnically diverse population to analyze glucose and insulin dynamics accounting for covariates are required to gain a clearer understanding of sex-mediated differences in the regulation of glucose metabolism.

## Sexual dimorphic interaction of β-cells

Previous rodent models of adult-onset diabetes mellitus, where β-cell failure is present, exhibit sexual dimorphic results, whereby females are protected to some degree from disease development (49). There are several hormones which modulate β-cell function, growth, and survival to adapt to metabolic stresses which appear to have sexual dimorphic traits. For example, gonadal estrogens have been shown to regulate β-cell function and survival. Previously, exogenous 17β-estradiol have shown to be protective against β-cell apoptosis from streptozotocin injections in adult male mice (49). Clinical studies have observed increased exogenous 17β-estradiol levels in pregnancies with a female fetus, however, this finding is not consistent (50).

Changes in islet growth and function have strong correlations with serum levels of placental lactogen and prolactin (51). Previously, placental lactogen has been shown to be decreased in maternal sera in pregnancies with a male fetus, however only significant in case of the umbilical levels (female = 25μg/L *vs.* male = 19μg/L) (52). Both lactogens aid to increase insulin secretion and β-cell proliferation, survival and mass, and lower threshold for glucose stimulated insulin secretion (7, 53). This mechanism is mediated through gene signaling in the β-cell via the prolactin receptor (PRLR) to increase serotonin synthesis. Activation of the PRLR induces a three-fold increase in the two isoforms, tryptophan hydroxylase 1 and 2, driving serotonin synthesis, storage, and co-secretion within insulin during pregnancy. Kim et al. (2010) demonstrated expression of tryptophan hydroxylase-1 and serotonin production increased in islets during pregnancy and following treatment with lactogens (54). Conversely, targeted inhibition of the serotonin synthesis reduced β-cell expansion leading to impaired glucose tolerance in pregnant mice without affecting insulin sensitivity (54).

**Figure 1:**
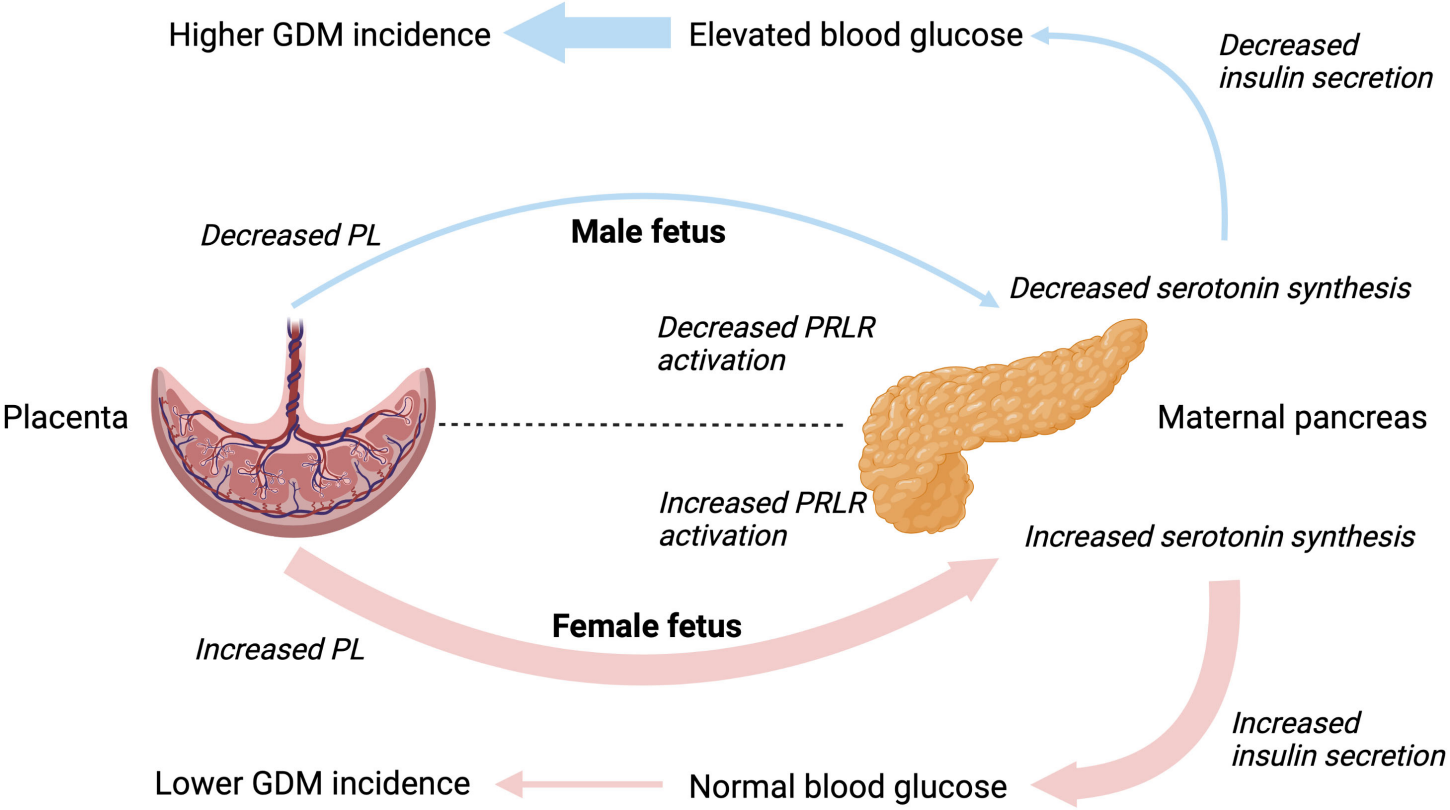
Hypothesized interactions between placental lactogen and maternal β-cell function driving the different incidence rates of gestational diabetes mellitus in male and female pregnancies. PL, placental lactogen; PRLR, prolactin receptor; GDM, gestational diabetes mellitus.

These findings may suggest any fetal-sex-specific association of GDM with a male fetus is driven by β-cell dysfunction rather than maternal insulin resistance. Overall, proposing carrying a male fetus might be associated with decreased maternal hormones necessary for β-cell survival and proliferation (**Figure 1**). These findings might explain further the spectrum of insulin sensitivity and secretion defects related to GDM described previously (1, 3). However, the association of fetal sex with GDM, although statistically significant, is of small magnitude and it could be speculated that fetal sex is of clinical relevance only when associated with other major risk factors, such as family history and/or genetic susceptibility to maternal hyperglycemia.

## Clinical management of maternal hyperglycemia incorporating fetal sex

There is little research incorporating the independent effects of fetal sex in the management of GDM. An initial report from the USA-based Maternal Fetal Medicine Network Unit randomized controlled trial concluded there was significant reduction in birth weight and accumulated fat mass associated with treatment of mild GDM in males which was not observed in female newborns (55). The reason for this differential treatment effect between sexes in mild GDM is not known. The authors of this paper suggest the observations may be linked back to the male fetus’ susceptibility to oxidative stress, as markers of oxidative stress play important roles in lipogenesis and fat accumulation. Therefore, treatment aimed at reducing oxidative stress are hypothesized to have a stronger effect in the male fetus. By contrast, in a follow-up study of the same cohort which examined children at age 5-10 years, Landon et al. (2015) concluded only female offspring of women treated for mild GDM exhibited glucometabolic benefits (56). Due to the lack of research in this area and disparity between early and later postnatal findings, there are no clear conclusions regarding clinical management of GDM based on fetal sex.

## Conclusion

Studies to date have provided inconsistent overall evidence for a sexual-dimoprhic pattern in the prevalence of GDM and subsequent postpartum risks. This includes the suggested increased risk of GDM in pregnancies carrying a male fetus, an increased risk of subsequent type 2 diabetes in GDM women carrying a female fetus, and more adverse outcomes for the male fetus in pregnancies complicated by pre-existing diabetes mellitus or GDM. These studies have not, however, been designed to study mechanism(s) and they lack granular patient level data. Further, most studies have been confounded by differences in the treatment of maternal hyperglycemia. Future well-designed studies focusing on underlying mechanism(s) are crucial to advance our understanding of the field, and guide both antenatal surveillance and treatment strategies.
